# Artemther-lumefantrine is partially effective for treating chronic multi-species malaria in Ugandan pre-school children

**DOI:** 10.1186/1475-2875-11-S1-P11

**Published:** 2012-10-15

**Authors:** Martha Betson, José C Sousa-Figueiredo, Sarah Clifford, Aaron Atuhaire, Moses Arinaitwe, Moses Adriko, Gerald Mwesigwa, Juma Nabonge, Narcis B Kabatereine, Colin J Sutherland, J Russell Stothard

**Affiliations:** 1 Liverpool School of Tropical Medicine, Liverpool L3 5QA, UK; 2 Faculty of Infectious and Tropical Diseases, London School of Hygiene and Tropical Medicine, London WCIE 6BT, UK; 3 Vector Control Division, Ministry of Health, Kampala, Uganda; 4 Vector Control Division, Mayuge District, Mayuge, Uganda

## Background

In Uganda artemether-lumefantrine (AL) is the first-line treatment for uncomplicated falciparum malaria. During a longitudinal study (SIMI project) investigating the dynamics of intestinal schistosomiasis and malaria in Ugandan lakeshore communities, a consistently high prevalence of malaria was found in young children (including many mixed infections of *Plasmodium falciparum* with *P. malariae* and/or *P. ovale* sspp), despite use of AL for home-based management of malaria. As a first step to gather evidence on the effectiveness of AL in this setting, a community-based observational study was initiated in an area of intense malaria transmission.

## Materials and methods

The study was carried out in Bukoba village, Mayuge District. A group of children (W = 163) within the SIMI cohort was selected on the basis of a positive First Response rapid diagnostic test (RDT) result and microscopy-confirmed malaria. Children were actively followed up 7 and 17 days after baseline. Individuals who were RDT-positive on Day 17 were also followed up on Day 24. At each time-point, a blood smear archive was made and microscopy was performed two-three days after samples were taken. In addition, blood spots were collected onto Whatmann 3MM filter paper. Treatment decisions in the field were based on RDT results. All study participants were treated with AL at baseline. Children who were malaria positive by OptiMAL RDT on Day 7 were retreated with AL and those who were malaria positive on Day 17 were treated with oral quinine. All children were tested for *Schistosoma mansoni* and soil-transmitted helminth infections at baseline and treated with praziquantel and albendazole.

Genomic DNA was extracted from blood spots using chelex and real-time PCR diagnosis of *Plasmodium* species was performed. To distinguish between recrudescent and new infections, genotyping of merozoite surface proteins (*msp1* and *msp2*) and glutamate-rich protein (*glurp*) was carried out.

## Results

Forty children (26.3%) were microscopy-positive for malaria on Day 7 and 33 (21.3%) on Day 17. After genotyping, 33 (21.9%) and 17 (11.7%) children were shown to have recrudescent infections on Days 7 and 17, respectively. Of the 28 children who had received two consecutive AL treatments, 11 were microscopy positive on Day 17.

Multi-species *Plasmodium* infections were common, with 41.1% of children positive for *P. falciparum/P. malariae*, 9.2% positive for *P. falciparum/P. ovale* sspp. and 8.0% for all three species at baseline. By real-time PCR 39.9% of those children infected with falciparum malaria at baseline were *P. falciparum* positive at Day 17 and 9.2% of those who were infected with *P. malariae* at baseline were *P. malariae* positive at Day 17. On Day 24, after two or three consecutive anti-malarial treatments, 10 children were infected with *P. falciparum* and two with *P. malariae.*

## Conclusions

Our results suggest that AL may not be as effective as previously thought for treatment of malaria at a community-based level in Uganda and that further more formalised efficacy studies of this drug in high transmission settings are required, particularly in areas where mixed-species malaria infections are common and where mass administration of anthelminthic drugs is being carried out.

